# Draft Genome Sequence of *Pseudomonas* sp. Strain CCA1, Isolated from Leaf Soil

**DOI:** 10.1128/genomeA.01371-16

**Published:** 2016-12-08

**Authors:** Hironaga Akita, Zen-ichiro Kimura, Tamotsu Hoshino

**Affiliations:** a Research Institute for Sustainable Chemistry, National Institute of Advanced Industrial Science and Technology (AIST), Kagamiyama, Higashi-Hiroshima, Hiroshima, Japan; b Department of Civil and Environmental Engineering, National Institute of Technology, Kure College, Aga, Minami, Kure, Hiroshima, Japan; c Bioproduction Research Institute, National Institute of Advanced Industrial Science and Technology (AIST), Tsukisamu-Higashi, Toyohira-ku, Sapporo, Japan

## Abstract

*Pseudomonas* sp. strain CCA1 was isolated from leaf soil collected in Higashi-Hiroshima City in Hiroshima Prefecture, Japan. Here, we present a draft genome sequence of this strain. The genome consists of 24 contigs for a total of 6,993,992 bp, 8,917 predicted coding sequences, and a GC content of 67.2%.

## GENOME ANNOUNCEMENT

Lignin is an aromatic polymer and the second most abundant organic polymer on Earth (1). Thus, effective utilization of lignin could support second-generation biofuel production from lignocellulosic biomass (2). *Phanerochaete chrysosporium*, *Rhodococcus erythropolis*, and *Streptomyces coelicolor* are able to assimilate lignin as a carbon source (3). However, methods for their culture are intricate and their growth is relatively slow, making them unsuitable for industrial production of second-generation biofuels. We therefore screened for lignin-degrading bacteria with rapid growth rates and high capacities for lignin degradation. This led to the isolation of *Pseudomonas* sp. strain CCA1 (strain number HUT-8136) from leaf soil (4).

After *Pseudomonas* sp. strain CCA1 was aerobically cultured overnight at 37°C in nutrient broth (Kyokuto), the genomic DNA was extracted and purified using an illustra bacteria genomicPrep mini spin kit (GE Healthcare) according to the manufacturer’s instructions. The purity and concentration of the genomic DNA and double-stranded DNA were measured using a NanoDrop (Thermo Scientific) and a Quant-iT dsDNA BR Assay Kit (Invitrogen), respectively. After fragmenting the genomic DNA (7.8 µg) into approximately 20-kb pieces using a g-TUBE (Covaris), the resultant fragments were ligated to SMRTbell sequencing adapters using an SMRTbell Template Prep Kit 1.0 (Pacific Biosciences), which yielded the SMRTbell libraries. The library size was measured using an Agilent 2200 TapeStation (Agilent Technologies). The SMRTbell libraries were then bound to polymerases and sequencing primers using a DNA/Polymerase Binding Kit P6 v2 (Pacific Biosciences), which yielded the sequencing templates. After the concentration of the sequencing templates was calculated using Binding Calculator Version 2.3.1.1 (Pacific Biosciences), the templates were bound to MagBeads using a MagBead Kit (Pacific Biosciences) and loaded onto single-molecule real-time (SMRT) cells 8 Pac V3 (Pacific Biosciences). The sequencing was performed using the PacBio RS II platform (Pacific Biosciences). The raw data included 63,138 reads at 125-fold coverage and were assembled *de novo*, using SMRT Analysis v2.3.0 (Pacific Biosciences) (5) to filter the subreads. The genome sequence consisted of 6,993,992 bp with a GC content of 67.2%. The assembly generated 24 contigs with an *N*_50_ contig size of 6,935,186 bp. Genome annotation was performed using CRITICA (6) and Glimmer2 (7), and 8,917 predicted coding sequences were identified. In addition, 68 tRNA genes and 17 rRNA genes were identified using tRNAScan-SE (8) and BLASTN (9), respectively.

### Accession number(s).

The nucleotide sequence and annotation data for the *Pseudomonas* sp. strain CCA1 draft genome have been deposited in DDBJ/EMBL/GenBank under accession numbers BDGS01000001 to BDGS01000024.
